# Testing Gait with Ankle-Foot Orthoses in Children with Cerebral Palsy by Using Functional Mixed-Effects Analysis of Variance

**DOI:** 10.1038/s41598-017-11282-1

**Published:** 2017-09-11

**Authors:** Bairu Zhang, Richard Twycross-Lewis, Heiko Großmann, Dylan Morrissey

**Affiliations:** 1 School of Mathematical Sciences, Queen Mary University of London, London, E1 4NS UK; 2 Sports and Exercise Medicine, Queen Mary University of London, London, E1 4DG UK; 3 Fakultät für Mathematik, Otto-von-Guericke-Universität Madgeburg, 39106 Madgeburg, Germany; 4 Physiotherapy Department, Barts Health NHS Trust, London, E1 UK

## Abstract

Existing statistical methods extract insufficient information from 3-dimensional gait data, rendering clinical interpretation of impaired movement patterns sub-optimal. We propose an alternative approach based on functional data analysis that may be worthy of exploration. We apply this to gait data analysis using repeated-measurements data from children with cerebral palsy who had been prescribed fixed ankle-foot orthoses as an example. We analyze entire gait curves by means of a new functional F test with comparison to multiple pointwise F tests and also to the traditional method - univariate repeated-measurements analysis of variance of joint angle minima and maxima. The new test maintains the nominal significance level and can be adapted to test hypotheses for specific phases of the gait cycle. The main findings indicate that ankle-foot orthoses exert significant effects on coronal and sagittal plane ankle rotation; and both sagittal and horizontal plane foot rotation. The functional F test provided further information for the stance and swing phases. Differences between the results of the different statistical approaches are discussed, concluding that the novel method has potential utility and is worthy of validation through larger scale patient and clinician engagement to determine whether it is preferable to the traditional approach.

## Introduction

Functional data analysis^1–4^ (FDA) is an umbrella term for statistical methods that are applicable when the measured responses are not numbers but functions of time, space or some other domain. When the domain is time, responses are typically represented by curves. Data of this kind arise commonly, for instance, in laboratory settings where measurements can be taken almost continuously at densely spaced time points. Human gait research is an important clinical and experimental setting where functional data are collected to guide intervention decisions. In this field, the curves of interest often depict the rotation of a joint during a stride, across a standardized time interval between one foot contact to the next by the same foot.

Early work on FDA for human gait data developed methods for estimating mean and covariance functions^5^ and for calculating prediction regions for entire curves^6^, and applied these to samples of curves that were collected on healthy children. Other researchers^1, 7–9^ used the data^5, 6^ to illustrate various types of FDA techniques, including functional principal components analysis, functional canonical correlation analysis and functional regression. Data from an experiment^10^ where volunteers were stepping in place have served as an example to demonstrate the use of functional analysis of variance^10–12^ (ANOVA) for investigating the effects of different orthosis conditions on moments at the knee. Despite these examples and the fact that the FDA approach appears to be particularly useful for studying gait curves^13^, only a few gait studies^14–16^ have used FDA to investigate questions of genuine clinical interest. Moreover, only relatively basic FDA methods^1^ seem to have been used in clinical applications. Possible reasons for this are that more advanced FDA techniques are less widely known and theoretically and computationally more complex.

This paper proposes a new method of functional mixed-effects ANOVA for studying gait data of children with cerebral palsy, who have abnormal gait patterns leading to fixed ankle-foot orthoses prescription. We analyze gait curves that were collected from a repeated-measurements design in which barefoot walking preceded walks with ankle-foot orthoses. The use of ankle-foot orthoses to control movements of patients with cerebral palsy has a long history^17^ and the main purpose of an ankle-foot orthosis is to enhance function by improving motion of lower limb body segments during the gait cycle^18^. Ambulatory function, such as walking ability, balance and stability, is qualitatively assessed by physiotherapists. Quantitative gait analysis occurs in specialist centres when critical decisions, such as orthosis prescription or operative intervention consideration, are being made and require interpretation of complex data sets. In this study we investigate the effects of ankle-foot orthoses on quantitatively measured lower limb 3-dimensional joint rotation during gait, also known as the study of kinematics^19^.

The effects of ankle-foot orthoses on kinematic gait data of patients with cerebral palsy have previously been examined in several studies^20–26^. However, in these studies gait curves were not treated as analyzable entities and kinematic parameters reflecting particular characteristics of the curves were used as the response variable in a univariate analysis. Examples of such response parameters include joint rotation values at specific gait events such as heel strike or toe-off^23–25^, maximal or minimal rotation during the gait cycle or midpoints of identifiable gait phases such as stance^21, 23–25^, and mean and range of rotation for the whole or parts of the gait cycle^26^. Integrating findings that are based on different kinds of parameters is not always straightforward. Moreover, considering the effects of ankle-foot orthoses throughout the gait cycle has been recommended to ensure important findings are not neglected^27^ and the FDA approach would meet this criterion.

In what follows, we present a novel application of FDA to entire gait curves from children with cerebral palsy which allows us to test the effect of ankle-foot orthoses while accounting for the repeated-measurements nature of the data. Repeated measurements are modeled by a special case of a functional mixed-effects model^28^, although we avoid the complex computations that are involved when the model is fitted in a Bayesian manner^28–31^. We propose a new functional F test which integrates information over the whole gait cycle and compare the results of its application with those of multiple pointwise F tests that are performed at equally spaced time points in the gait cycle. In addition, the functional F test is also compared with the traditional univariate F test in a one-way repeated-measurements ANOVA^32^, which is commonly used in gait studies^22–24^.

The main purpose of this study is to explore suitable FDA techniques that can be applied to complex data by developing a better tool for testing functional data collected from repeated-measurements experiments, in which multiple curves are collected from each subject. In the specific case considered here, the functional F test is applied to examine the global effects of ankle-foot orthoses at a group level to guide clinicians. This application will also facilitate utilization of functional F tests in other domains with similar data.

Tests for functional mixed-effects models have only been considered in a few reports^33, 34^. The new test generalizes previous work on functional F tests^3, 35^ for independent curves, to repeated measurements in which the curves are correlated. The proposed functional F test preserves the nominal significance level, whereas the multiple testing approach based on pointwise tests is subject to a potentially large familywise error probability. Moreover, the functional test can be easily modified to test the effect of the orthoses for different well-defined phases of the gait cycle^36, 37^, thereby enabling the researcher to tailor the analysis to specific research questions. We report corresponding results for the stance and swing phases respectively.

## Method

### Data collection

The present study was performed in accordance with the ethical guidelines of the Declaration of Helsinki and was approved by East London NHS Research Ethics Committee (Ethics REF 09/H0806/56). Written informed assent and consent, from all children and parents respectively, was collected.

**Figure 1:**
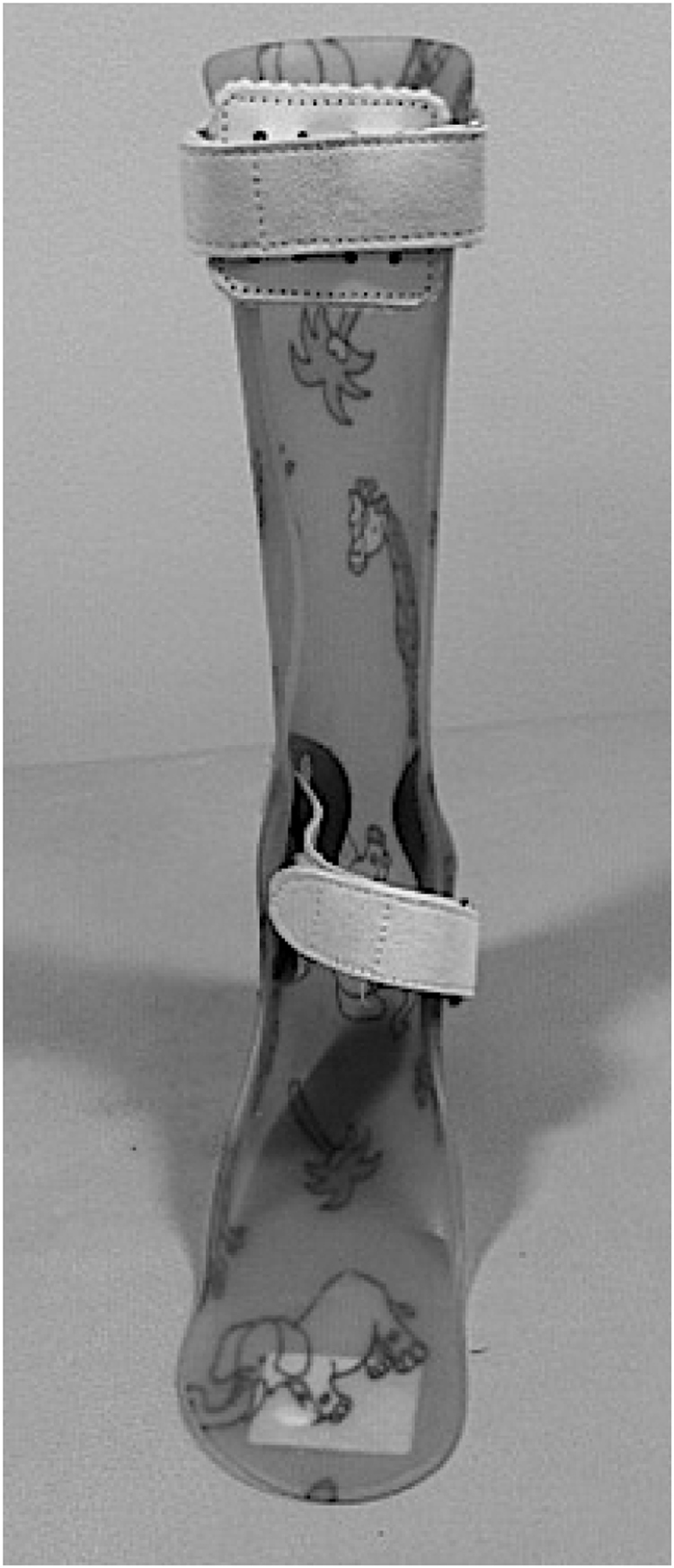
Bespoke, fixed ankle-foot orthoses for children with cerebral palsy.

Time-dense gait data were collected from fourteen children (mean age 12.3 ± 2.88*years*, mean height 1.44 ± 0.15 *m*, mean weight 39.57 ± 11.78 *kg*) at the Human Performance Laboratory, Queen Mary University of London. All recruited children had been diagnosed with spastic cerebral palsy and prescribed fixed ankle-foot orthoses (see Fig. 1) for a minimum of six months. Children were initially assessed by a paediatric orthopaedic consultant and only included if they were independently ambulatory and considered to have sufficient muscular endurance for gait measurements. This study was designed after the clinical assessment had taken place, and hence we were not approved to access the medical records of patients nor was the direct clinical interpretation our primary focus. Nonetheless, we consulted the physiotherapist who accompanied patients for data collection and she confirmed that most recruited children were classified to the Gross Motor Function Classification System (GMFCS)^38^ level 2 and 3 and few children were classified at level 4.

Data collection followed the commonly used protocol^20, 25, 39^ whereby each patient was instructed to perform a series of walks both barefoot and wearing ankle-foot orthoses placed within shoes. Order was not randomised and barefoot walking was conducted first^20, 25^. While walking with ankle-foot orthoses, patients were shod, owing to the importance of footwear in the orthotic prescription that ankle-foot orthoses modify kinematics of segments only with appropriate footwear^27^. More specifically, anthropometric information, including pelvic width and depth and bilateral knee and ankle width, was obtained. Then kinetic data were collected while the patient walked barefoot at a self selected pace along a 6-meter walkway with two ground embedded force plates (Type 9281B Multicomponent Force Plate, Kistler Instruments Ltd, Winterthur, Switzerland) that measured 3-dimensional ground reaction force. After 10–20 walks, patients then repeated walking tests whilst wearing their ankle-foot orthoses over the same force plates.

Kinematic data were collected using four 3D Cartesian Optoelectric Dynamics Anthropometer systems (Charnwood Dynamics, Rotheley, Leicestershire, UK) that were placed at distances of 2–3 meters from the force plates, oblique to the centre of the laboratory in order to create a data collection volume. A modified Helen Hayes marker set protocol^19^ was used, whereby active infra-red markers were placed bilaterally on the anterior sacro-iliac spine (ASIS); posterior sacro-iliac spine (PSIS); lateral epicondyle of the knee and the lateral malleolus; lateral aspect of the calcaneous and the 5th metatarsal. Instrumented marker wand sets were also placed superior and inferior to the knees. Joint centers for the pelvis, hips, knees and ankles were calculated using Codamotion Analysis software (version 6.76.2-CX1/mpx30, Charnwood Dynamics, Rotheley, Leicestershire, UK) based on subject specific anthropometric data. Gait events in each trial, from initial contact to toe off to the following initial contact, were marked using the vertical component of ground reaction force and velocity of the calcaneous marker for both the ipsilateral and contralateral limbs. Standardized gait graphs were then extracted by analyzing the kinematic data offline using Matlab (version 2009a, The Mathworks, Natick, MA, USA).

### Statistical model

For testing the effect of ankle-foot orthoses, we consider the functional mixed-effects ANOVA model

1 $${y}_{ijk}(t)=\mu (t)+{\alpha }_{i}(t)+{\beta }_{j(i)}(t)+{\gamma }_{k}(t)+{\varepsilon }_{ijk}(t),\quad t\in {\mathscr{T}}=\mathrm{[0},\mathrm{1],}$$

where *μ*(*t*) is the overall mean function; *α*_*i*_(*t*) for *i* = 1, …, 14 is the *i* th subject-specific random effect; *β*_*j*(*i*)_(*t*) for *j* = 1, 2 is the random effect for the *j* th lower limb nested within the *i* th subject; *γ*_*k*_(*t*) for *k* = 1, 2 is the fixed effect for wearing (or respectively not wearing) ankle-foot orthoses and *ε*_*ijk*_(*t*) is the error term. The total number of response curves ${y}_{ijk}(t)$ is *n* = 56. The random effect terms *α*_*i*_(*t*), ${\beta }_{j(i)}(t)$ and the error term ${\varepsilon }_{ijk}(t)$ are assumed to be independent zero-mean Gaussian processes, each with its own covariance function. More specifically, by using the generic notation $GP\mathrm{(0,}\,\theta )$ for a zero-mean Gaussian process with covariance function $\theta \equiv \theta (s,\,t)$, it is assumed that ${\alpha }_{i}(t)\sim GP\mathrm{(0,}\,{\theta }_{a})$, ${\beta }_{j(i)}(t)\sim GP\mathrm{(0,}\,{\theta }_{b})$ and ${\varepsilon }_{ijk}(t)\sim GP\mathrm{(0,}\,{\theta }_{e})$ for $i=1,\,\ldots ,\,14$, *j* = 1, 2 and *k* = 1, 2, all independent.

Since for all curves *y*_*ijk*_(*t*), *μ*(*t*), *α*_*i*_(*t*), *β*_*j*(*i*)_(*t*), *γ*_*k*_(*t*) and *ε*_*ijk*_(*t*) we use the same time points of the gait cycle, at every fixed time point $t\in {\mathscr{T}}$ equation (1) can be regarded as the model equation of a univariate repeated-measurements ANOVA model. Hence, at every fixed *t*, pointwise sums of squares *SS*(*t*) and expected mean squares *EMS*(*t*) for the different terms in the model can be calculated as for the univariate model. However, when *t* traverses the whole gait cycle, both *SS*(*t*) and *EMS*(*t*) become functions of *t*, which we refer to as the functional sum of squares and the functional expected mean squares respectively.

The pointwise ANOVA table for fixed $t\in {\mathscr{T}}$ is presented in Table 1 in which the various means are given by

2 $${\bar{y}}_{\mathrm{...}}(t)=\frac{1}{n}\sum _{i,j,k}{y}_{ijk}(t),\quad {\bar{y}}_{i\mathrm{..}}(t)=\frac{1}{2\times 2}\sum _{j,k}{y}_{ijk}(t),\quad {\bar{y}}_{ij\mathrm{.}}(t)=\frac{1}{2}\sum _{k}{y}_{ijk}(t)\quad \,{\rm{and}}\,\quad {\bar{y}}_{\mathrm{..}k}(t)=\frac{1}{14\times 2}\sum _{i,j}{y}_{ijk}(t\mathrm{).}$$

**Table 1:** Pointwise ANOVA table with “AFO” indicating the factor for wearing/not wearing ankle-foot orthoses.

	Degrees of freedom	*SS*(*t*)	*EMS*(*t*)
mean	1	$\sum _{i=1}^{14}\sum _{j=1}^{2}\sum _{k=1}^{2}{\bar{y}}_{\mathrm{...}}{(t)}^{2}$	
subjects	13	$\sum _{i=1}^{14}\sum _{j=1}^{2}\sum _{k=1}^{2}{\{{\bar{y}}_{i\mathrm{..}}(t)-{\bar{y}}_{\mathrm{...}}(t)\}}^{2}$	
limbs	14	$\sum _{i=1}^{14}\sum _{j=1}^{2}\sum _{k=1}^{2}{\{{\bar{y}}_{ij\mathrm{.}}(t)-{\bar{y}}_{i\mathrm{..}}(t)\}}^{2}$	
AFO	1	$\sum _{i=1}^{14}\sum _{j=1}^{2}\sum _{k=1}^{2}{\{{\bar{y}}_{\mathrm{..}k}(t)-{\bar{y}}_{\mathrm{...}}(t)\}}^{2}$	$14\times 2\times \sum _{k=1}^{2}{\gamma }_{k}{(t)}^{2}+{\theta }_{e}(t,t)$
residual	27	by subtraction	${\theta }_{e}(t,t)$
Total	56	$\sum _{i=1}^{14}\sum _{j=1}^{2}\sum _{k=1}^{2}{y}_{ijk}{(t)}^{2}$	

The breakdown of the total sum of squares in the table into sums of squares for the different sources of variation is valid at every point *t* and, hence, also for the functional sums of squares. Equation (1) together with the usual constraint ${\gamma }_{1}(t)+{\gamma }_{2}(t)=0$ for all $t\in {\mathscr{T}}$ implies that for every *t* the expected mean squares for the sums of squares $S{S}_{AFO}(t)={\sum }_{i,j,k}{\{{\bar{y}}_{\mathrm{..}k}(t)-{\bar{y}}_{\mathrm{...}}(t)\}}^{2}$ and $S{S}_{residual}(t)={\sum }_{i,j,k}{\{{y}_{ijk}(t)-{\bar{y}}_{ij\mathrm{.}}(t)-{\bar{y}}_{\mathrm{..}k}(t)+{\bar{y}}_{\mathrm{...}}(t)\}}^{2}$ come out as shown in Table 1.

The ANOVA in Table 1 provides the basis for testing if ankle-foot orthoses have an effect. Both types of test, the pointwise F tests and the functional F test, use the sums of squares in the table. However, whereas the pointwise F tests also use the degrees of freedom in the table in order to assess the significance of the results, this is not the case for the functional F test.

### Pointwise F tests

The effect of ankle-foot orthoses may be tested by adopting a multiple testing approach. This amounts to performing a series of separate F tests of

3 $${H}_{0}:{\gamma }_{1}({t}_{\ell })={\gamma }_{2}({t}_{\ell })\quad {\rm{versus}}\quad {H}_{1}:{\gamma }_{1}({t}_{\ell })\ne {\gamma }_{2}({t}_{\ell })$$

at each of *m* equally spaced points ${t}_{\ell }\in {\mathscr{T}}$, $\ell =1,\ldots ,m$, in the gait cycle. We refer to these tests as pointwise F tests. The test statistic of the pointwise F test at ${t}_{\ell }$ and its distribution under the null hypothesis *H*_0_ are given by

4 $$F({t}_{\ell })=\frac{S{S}_{AFO}({t}_{\ell })/1}{S{S}_{residual}({t}_{\ell })/27}\sim F(1,\,27).$$

The resulting values $F({t}_{\ell })$ are plotted against ${t}_{\ell }$ for $\ell =1,\ldots ,m$ and can be assessed for statistical significance at every time point.

Pointwise F tests do not take the functional nature of the data into account. Moreover, this approach faces the usual problems surrounding multiple testing^40^. In particular, the familywise error probability can be much higher than the nominal significance level of the individual tests as will be illustrated later.

### Functional F test

As an alternative to multiple testing with pointwise F tests we propose a new functional F test. The functional F test summarizes information across the whole gait cycle by integrating the functional sums of squares *SS*_*AFO*_(*t*) and *SS*_*residual*_(*t*) over ${\mathscr{T}}$ and uses the ratio of the integrals as the test statistic. Contrary to the pointwise F tests, the hypotheses tested by the functional F test refer to the whole curves ${\gamma }_{k}(t)$, *k* = 1, 2, in Equation (1). More specifically, the testing problem is given by

5 $${H}_{0}:{\gamma }_{1}(t)={\gamma }_{2}(t)\,{\rm{for}}\,{\rm{all}}\,t\in {\mathscr{T}}\quad {\rm{versus}}\quad {H}_{1}:{\gamma }_{1}(t)\ne {\gamma }_{2}(t)\,{\rm{for}}\,{\rm{some}}\,t\in {\mathscr{T}}\mathrm{.}$$

The null hypothesis *H*_0_ states that the two functions *γ*_1_(*t*) and *γ*_2_(*t*) are equal, whereas the alternative hypothesis *H*_1_ says that they are different. In order to test *H*_0_ against *H*_1_, the functional F test uses the single statistic

6 $$ {\mathcal F} =\frac{{\int }_{{\mathscr{T}}}S{S}_{AFO}(t)dt/1}{{\int }_{{\mathscr{T}}}S{S}_{residual}(t)dt/27}.$$

Under the null hypothesis *H*_0_ of no effect, by using arguments similar to the case of independent curves^3, 35^, the distributions of the integrated sums of squares in (6) can be shown to be mixtures of independent chi square distributions^34^. More precisely, under *H*_0_ it holds that

7 $$\begin{array}{ll}{\int }_{{\mathscr{T}}}S{S}_{AFO}(t)dt & ={\int }_{{\mathscr{T}}}\sum _{i,j,k}{\{{\bar{\varepsilon }}_{\mathrm{..}k}(t)-{\bar{\varepsilon }}_{\mathrm{...}}(t)\}}^{2}dt\sim \sum _{r=1}^{\infty }{\lambda }_{r}{\chi }_{1}^{2},\\ {\int }_{{\mathscr{T}}}S{S}_{residual}(t)dt & ={\int }_{{\mathscr{T}}}\sum _{i,j,k}{\{{\varepsilon }_{ijk}(t)-{\bar{\varepsilon }}_{ij\mathrm{.}}(t)-{\bar{\varepsilon }}_{\mathrm{..}k}(t)+{\bar{\varepsilon }}_{\mathrm{...}}(t)\}}^{2}dt\sim \sum _{r=1}^{\infty }{\lambda }_{r}{\chi }_{27}^{2}\mathrm{.}\end{array}$$

In (7), the means ${\bar{\varepsilon }}_{\mathrm{...}}(t)$, ${\bar{\varepsilon }}_{ij\mathrm{.}}(t)$ and ${\bar{\varepsilon }}_{\mathrm{..}k}(t)$ of the random errors ${\varepsilon }_{ijk}(t)$ in equation (1) are computed like the corresponding means of the responses in (2). Moreover, ${({\lambda }_{r})}_{r\ge 1}$ is the sequence of eigenvalues of the covariance operator^4^ associated with the covariance function ${\theta }_{e}(s,t)$ and ${\sum }_{r=1}^{\infty }{\lambda }_{r}{\chi }_{1}^{2}$ denotes the distribution of a mixture of independent random variables, each of which has a chi square distribution with $1$ degree of freedom, while ${\sum }_{r\mathrm{=1}}^{\infty }{\lambda }_{r}{\chi }_{27}^{2}$ represents a similar mixture of independent random variables, each having a chi square distribution with 27 degrees of freedom.

The sums of squares $S{S}_{AFO}(t)$ and $S{S}_{residual}(t)$ are independent for every $t\in {\mathscr{T}}$ and it can be shown that this property carries over to the integrals ${\int }_{{\mathscr{T}}}S{S}_{AFO}(t)dt$ and ${\int }_{{\mathscr{T}}}S{S}_{residual}(t)dt$. The same arguments^35^ as in the derivation of the functional F test for the functional linear fixed-effects-only model then show that under *H*_0_ the distribution of ${\mathcal F}$ in (6) can be approximated by an F distribution as follows

8 $$ {\mathcal F} \mathop{\sim }\limits^{approx\mathrm{.}}F(d{f}_{AFO},d{f}_{residual}),$$

with degrees of freedom equal to $d{f}_{AFO}=\frac{{({\sum }_{r=1}^{\infty }{\lambda }_{r})}^{2}}{{\sum }_{r=1}^{\infty }{\lambda }_{r}^{2}}$ and $d{f}_{residual}=27\times \frac{{({\sum }_{r=1}^{\infty }{\lambda }_{r})}^{2}}{{\sum }_{r=1}^{\infty }{\lambda }_{r}^{2}}$, respectively.

The practical application of the functional F test requires the approximation of the integrals in the numerator and denominator of ${\mathcal F}$ and also the approximate computation of the eigenvalues that are needed for calculating the degrees of freedom $d{f}_{AFO}$ and $d{f}_{residual}$. To this end, we adapt the approaches^3, 35^ that have been used for the case of independent curves and which essentially amount to turning the functional problem into a multivariate problem.

More precisely, the interval ${\mathscr{T}}=\mathrm{[0,}\,\mathrm{1]}$ representing the gait cycle is discretized by superimposing a fine grid of $m$ equally spaced points ${t}_{\ell }$, $\ell =\mathrm{1,}\ldots ,m$, where ${t}_{1}=0 < {t}_{2} < \ldots  < {t}_{m-1} < {t}_{m}=1$. The integral ${\int }_{{\mathscr{T}}}S{S}_{AFO}(t)dt$ is then approximated by the sum ${\sum }_{\ell \mathrm{=1}}^{m}S{S}_{AFO}({t}_{\ell })$. Likewise, as an approximation to the integral ${\int }_{{\mathscr{T}}}S{S}_{residual}(t)dt$ the sum ${\sum }_{\ell \mathrm{=1}}^{m}S{S}_{residual}({t}_{\ell })$ is used. In the calculation of $d{f}_{AFO}$ and $d{f}_{residual}$, the sequence ${({\lambda }_{r})}_{r\ge 1}$ of eigenvalues is replaced by $m$ estimated eigenvalues ${\hat{\lambda }}_{1},\ldots ,{\hat{\lambda }}_{m}$ which are obtained as the eigenvalues of the $m\times m$ matrix $\hat{\Sigma }=({\hat{\theta }}_{e}({t}_{\ell },{t}_{q}))$, where for $\ell ,q\mathrm{=1,}\ldots ,m$

9 $${\hat{\theta }}_{e}({t}_{\ell },{t}_{q})=\frac{\sum _{i,j,k}\{{y}_{ijk}({t}_{\ell })-{\bar{y}}_{ij\mathrm{.}}({t}_{\ell })-{\bar{y}}_{\mathrm{..}k}({t}_{\ell })+{\bar{y}}_{\mathrm{...}}({t}_{\ell })\}\{{y}_{ijk}({t}_{q})-{\bar{y}}_{ij\mathrm{.}}({t}_{q})-{\bar{y}}_{\mathrm{..}k}({t}_{q})+{\bar{y}}_{\mathrm{...}}({t}_{q})\}}{27}.$$

is an estimate of ${\theta }_{e}({t}_{\ell },{t}_{q})$. Hence, in practice the degrees of freedom of the approximate null distribution of ${\mathcal F}$ are calculated as $d{f}_{AFO}=\frac{{({\sum }_{\ell =1}^{m}{\hat{\lambda }}_{\ell })}^{2}}{{\sum }_{\ell =1}^{m}{\hat{\lambda }}_{\ell }^{2}}$ and $d{f}_{residual}=27\times \frac{{({\sum }_{\ell =1}^{m}{\hat{\lambda }}_{\ell })}^{2}}{{\sum }_{\ell =1}^{m}{\hat{\lambda }}_{\ell }^{2}}$. Simple linear algebra shows, that the sums in the numerator and denominator of $d{f}_{AFO}$ and $d{f}_{residual}$ can be calculated respectively as the sum of the diagonal elements of the matrix $\hat{{\rm{\Sigma }}}$ and the sum of the diagonal elements of ${\hat{{\rm{\Sigma }}}}^{2}=\hat{{\rm{\Sigma }}}\hat{{\rm{\Sigma }}}$. In what follows, we use the value *m* = 201 which corresponds to splitting the gait cycle into two hundred intervals of equal width.

An attractive feature of the functional F test is that the integration over ${\mathscr{T}}$ can be replaced with integration over subsets of ${\mathscr{T}}$. This opens up the possibility to test the effect of ankle-foot orthoses in specific phases of the gait cycle as will be illustrated below.

## Results

### Observational results

**Figure 2:**
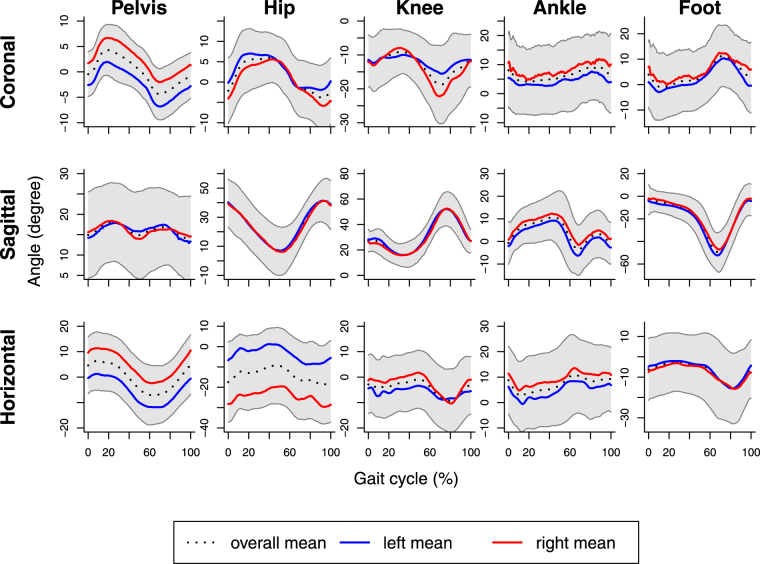
Barefoot walking in 14 children with cerebral palsy. Data are normalized to percentage (%) of the gait cycle, which starts from heel strike. Grey areas represent intervals [mean − s.d., mean + s.d.] at every point.

**Figure 3:**
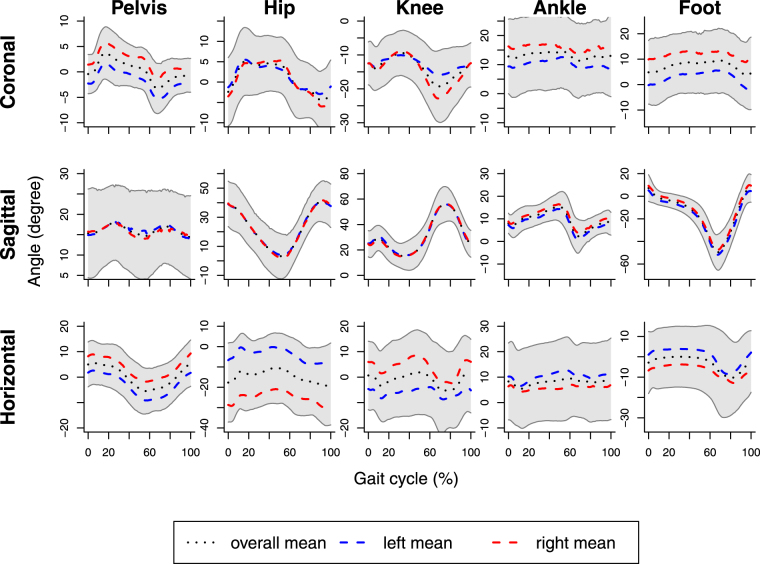
Gait with ankle-foot orthoses in 14 children with cerebral palsy. Data are normalized to percentage (%) of the gait cycle, which starts from heel strike. Grey areas represent intervals [mean − s.d., mean + s.d.] at every point.

Figures 2 and 3 show descriptive information for 3-dimensional segmental rotations for the lower body in 14 children with cerebral palsy during both barefoot and shod with ankle-foot orthoses walking respectively. In the figures, gait data are presented as a standardized gait report where columns show the pelvis, hip joint, knee joint, ankle joint and foot and rows are the coronal plane, sagittal plane and horizontal plane of rotation respectively. Differences can be seen in the overall kinematics with the application of the ankle-foot orthoses: there is an overall increase in maximal dorsiflexion from 10° to 15° of the ankle joint in the sagittal plane in gait with ankle-foot orthoses, however the magnitude of joint rotation does not change. Differences in the mean curves shown in Figs 2 and 3 are not immediately apparent.

### Pointwise F tests

**Figure 4:**
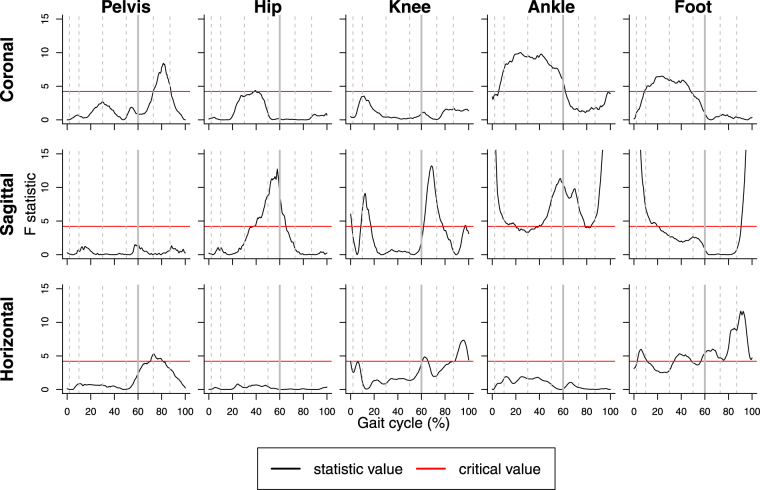
Multiple pointwise F tests at significance level *α* = 0.05 for effects of ankle-foot orthoses on segmental rotations. Grey dashed and solid lines are used to divide the whole gait cycle into phases: initial contact, loading response, mid-stance, terminal stance, pre-swing, initial swing, mid-swing and terminal swing (from left to right).

We first examined effects of ankle-foot orthoses on different segmental rotations by using the multiple pointwise F tests shown in Fig. 4. All individual tests used a signifiance level of *α* = 0.05. In the figure, ankle-foot orthoses have significant effects when the value of the F statistic exceeds the critical value, which is the same at all time points (${F}_{0.05}\mathrm{(1,}\,\mathrm{27)}=4.21$).

Effects of ankle-foot orthoses are more evident in the sagittal plane than in the coronal and horizontal planes. Generally, ankle-foot orthoses affect ankle joint and foot more than other segments, although there are also effects on the pelvis and the knee and hip joints. More specifically, in certain parts of the gait cycle ankle-foot orthoses have significant effects on pelvis in the coronal and horizontal planes; hip joint in the sagittal plane; knee joint in the sagittal and horizontal planes; ankle joint in the coronal and sagittal planes and foot in all three planes (Fig. 4). Moreover, for different segments significant effects of ankle-foot orthoses occur at different time points along the gait cycle conferring a temporal effect. Referring to Figs 2 and 3, we can see that for the pelvis in the coronal and horizontal planes, hip joint and ankle joint in the sagittal plane, ankle-foot orthoses have significant effects roughly around the minimal angles, whereas for the knee in the sagittal plane effects of ankle-foot orthoses tend to occur near maximal angles.

The nominal significance level of every individual pointwise F test is *α* = 0.05. However, in every panel of Fig. 4 many of those tests are performed on a grid of *m* = 201 points. Consequently, the familywise error rate^40^, which is the probability of at least one incorrect rejection of the null hypothesis, of this multiple testing procedure can be much higher than the nominal significance level if one looks at an interval rather than a single time point. For instance, ankle-foot orthoses affect hip rotation in the sagittal plane between 40–63% of the gait cycle (see Fig. 4). This section of the gait cycle contains approximately 46 grid points. At each of these points the probability of a type 1 error is 0.05. However, an approximate calculation under the simplifying (and surely not correct) assumption that the tests are independent shows that the familywise error rate for the 46 tests in the 40–63% interval of the gait cycle can be as high as 1 − (1 − 0.05)^46^ ≈ 0.90. This example illustrates that results of pointwise tests need to be interpreted with care, since the “significance” of the results may be overstated. Pointwise tests may suggest the presence of effects where there are actually none.

### Functional F test and univariate repeated-measurements analysis

The first part of Table 2 presents the functional F test for each of the segments and planes in Fig. 4. The values of the test statistic ${\mathcal F}$ and the corresponding p-values are shown in the second column of the table and the degrees of freedom of the approximate null distribution of ${\mathcal F}$ in the first column. The degrees of freedom are reported in the form $(d{f}_{AFO},d{f}_{residual})$ and depend on the actual data. At a significance level of $\alpha =0.05$, the functional F tests detect significant effects of ankle-foot orthoses on knee rotation in the sagittal plane $( {\mathcal F} =3.07,\,p=\mathrm{0.02)}$; ankle rotation in the coronal $( {\mathcal F} =5.95,\,p=\mathrm{0.02)}$ and sagittal $( {\mathcal F} =\mathrm{8.25,}\,p < \mathrm{0.01)}$ planes and foot rotation in the sagittal $( {\mathcal F} =\mathrm{3.61,}\,p=\mathrm{0.03)}$ and horizontal planes $( {\mathcal F} =5.27,\,p=\mathrm{0.01)}$. These results indicate that ankle-foot orthoses have significant effects on the overall motion of these segments over the whole gait cycle.

**Table 2:** Functional F tests for the whole gait cycle and F tests from univariate repeated-measurements (R-M) ANOVA for minimal and maximal angles.

		Functional F test		Univariate R-M ANOVA (F value (*p*.))	
		degrees of freedom	${\mathcal F}$ value (*p*.)	minimal angle	maximal angle
Pelvis	coronal	(2.12, 57.30)	1.89(0.16)	0.02(0.88)	0.16(0.69)
	sagittal	(1.41, 38.02)	0.38(0.61)	0.39(0.54)	0.02(0.89)
	horizontal	(1.77, 47.83)	1.61(0.21)	0.58(0.07)	0.51(0.48)
Hip	coronal	(2.77, 74.76)	0.98(0.40)	0.02(0.88)	0.24(0.63)
	sagittal	(2.37, 63.99)	2.53(0.08)	7.21(0.01)*	0.48(0.49)
	horizontal	(1.29, 34.98)	0.18(0.74)	0.08(0.77)	0.09(0.77)
Knee	coronal	(1.66, 44.82)	1.04(0.35)	1.06(0.31)	1.00(0.33)
	sagittal	(3.53, 95.36)	3.07(0.02)*	2.08(0.16)	3.42(0.08)
	horizontal	(1.38, 37.39)	2.60(0.10)	0.26(0.61)	8.02(<0.01)*
Ankle	coronal	(1.12, 30.23)	5.95(0.02)*	6.85(0.01)*	2.71(0.11)
	sagittal	(1.42, 38.44)	8.25(<0.01)*	21.1(<0.01)*	4.15(0.05)*
	horizontal	(1.21, 32.72)	0.87(0.38)	2.45(0.13)	0.95(0.34)
Foot	coronal	(1.15, 30.96)	3.10(0.08)	2.85(0.10)	0.68(0.42)
	sagittal	(2.29, 61.80)	3.61(0.03)*	2.49(0.12)	21.5(<0.01)*
	horizontal	(1.72, 46.39)	5.27(0.01)*	3.05(0.09)	7.09(0.01)*

Degrees of freedom for the univariate R-M ANOVA are (1, 27) for all segments and *indicates significance at the 0.05 significance level.

The second part of Table 2 reports results of a univariate repeated-measurements ANOVA with minimal angle as the response variable in the third column of the table, and corresponding results for maximal angle as the response variable in the fourth column. The F values in the table are computed as for the pointwise F test with the only modification that all squared differences that enter the sums of squares in Table 1 are calculated at the time points of the gait cycle at which the minimum (respectively maximum) angles occur. These time points do vary within and between patients. For both response variables, the null distribution of the test statistic is an $F\mathrm{(1},\mathrm{27)}$ distribution and thus the same as for the pointwise F test.

With significance level *α* = 0.05 as before, the F tests from the univariate repeated-measurements ANOVA detect effects of ankle-foot orthoses on minimal ankle angle in the coronal and sagittal planes, which agrees with the results of the functional F test, and, contrary to the functional F test, an effect on minimal hip angle in the sagittal plane. For maximal angle, the F tests from the univariate repeated-measurements ANOVA find significant effects on foot in the sagittal and horizontal planes as well as on ankle in the sagittal plane and these results are again in agreement with those of the functional F tests. Moreover, the repeated-measurements F test detects an effect on maximal knee angle in the horizontal plane where the corresponding functional F test for the whole gait cycle is not significant. Contrary to the functional F test, the repeated-measurements F tests with both minimal and maximal angle as the response do not detect a significant effect of ankle-foot orthoses on the knee joint in the sagittal plane.

### Functional F test for gait phases

Besides the whole gait cycle, we are also interested in effects of ankle-foot orthoses during the stance and swing phases separately. In normal gait, the stance phase accounts for the first 60% of the gait cycle and is defined as the period when the foot is in contact with the ground. Conversely, the swing phase accounts for approximately 40% of the gait cycle and is defined as the period when the foot does not have contact with the ground and is propelled forward ready for the next step^41^. This can also be seen in Fig. 4, where the stance and swing phases are divided by grey solid lines.

In order to perform functional F tests for these phases, it is only necessary to replace the interval ${\mathscr{T}}=\mathrm{[0,1]}$ in the formula for ${\mathcal F}$ in (6) with appropriate subintervals. For the stance phase we replace ${\mathscr{T}}$ with ${{\mathscr{T}}}_{1}=\mathrm{[0},\mathrm{0.6]}$ and for the swing phase we use ${{\mathscr{T}}}_{2}=\mathrm{(0.6},\mathrm{1]}$. Previous comments regarding the discretization of the interval ${\mathscr{T}}$ apply analogously to ${{\mathscr{T}}}_{1}$ and ${{\mathscr{T}}}_{2}$. For simplicity, we continue to denote the resulting test statistics by ${\mathcal F}$.

Results for the stance and gait phases are shown in Table 3. For significance level *α* = 0.05, ankle-foot orthoses have significant effects during the stance phase on hip rotation in the sagittal plane, ankle rotation in the coronal and sagittal planes, as well as on foot rotation in all three planes. In the swing phase, there are significant effects on knee rotation in the sagittal and horizontal planes, ankle rotation in the sagittal plane and foot rotation in the horizontal plane.

**Table 3:** Functional F tests for stance and swing phases of gait cycle with * indicating significance at 0.05 significance level.

		Stance phase ${\mathcal F}$ value (*p*.)	Swing phase ${\mathcal F}$ value (*p*.)
Pelvis	coronal	1.10(0.33)	3.12(0.07)
	sagittal	0.31(0.65)	0.49(0.54)
	horizontal	0.55(0.54)	3.26(0.07)
Hip	coronal	1.49(0.24)	0.26(0.75)
	sagittal	3.46(0.05)*	1.52(0.23)
	horizontal	0.28(0.65)	0.07(0.86)
Knee	coronal	1.09(0.33)	1.01(0.35)
	sagittal	1.35(0.27)	4.51(0.01)*
	horizontal	1.59(0.22)	4.11(0.04)*
Ankle	coronal	8.19(<0.01)*	2.23(0.14)
	sagittal	7.06(<0.01)*	9.80(<0.01)*
	horizontal	1.19(0.29)	0.29(0.62)
Foot	coronal	4.65(0.04)*	0.35(0.58)
	sagittal	4.61(0.03)*	2.80(0.08)
	horizontal	4.03(0.05)*	6.85(<0.01)*

### Comparison of statistical results

As can be seen from Fig. 4 and Table 2, all three methods of analysis consistently detect statistically significant effects of ankle-foot orthoses on the ankle joint in the coronal and sagittal planes and on the foot in the sagittal and horizontal planes. With the repeated-measurements ANOVA the former effects are significant when one looks at minimal angles (for maximal angles significance occurs only in the sagittal plane), while the latter effects are only significant for maximum angles. These results are also corroborated by the functional F tests for the stance and swing phases in Table 3.

In addition to these unequivocal findings, the pointwise F tests detect significant results for certain parts of the gait cycle where the functional F test for the whole gait cycle and the repeated-measurements ANOVA do not show significant effects. In particular, only the pointwise F tests find effects on the pelvis in the coronal and horizontal planes and on the foot in the coronal plane. For the pelvis in the horizontal plane, the significant pointwise F tests at around 80% of the gait cycle may be regarded as a false rejection due to the increased familywise error probability of the multiple testing approach or may be attributed to potential effects on minimal angles during the swing phase, although the corresponding p-values in Tables 2 and 3 are equal to *p* = 0.07 in both cases. Similarly, for the pelvis in the coronal plane, the visual impression from Fig. 4 is supported by the p-value of *p* = 0.07 of the corresponding functional F test for the swing phase (Table 3). A similar statement applies to the foot in the coronal plane and the corresponding functional F test for the stance phase.

By looking at the sagittal plane for the hip and knee joints in Fig. 4 one can see that the pointwise F tests detect significant effects just before and just after the point of transition from the stance to the swing phase. For the hip joint, these effects are also identified by the repeated-measurements ANOVA on minimal angles (Table 2) and the functional F test for the stance phase (Table 3). For the knee, the effect in the sagittal plane is detected by the functional F test for the swing phase and also by the functional F test for the whole gait cycle, although changes of the angles at around 20% may also have contributed to the latter result. For the knee joint in the horizontal plane the pointwise F tests signal some effect toward the end of the gait cycle, and this effect is also detected by the repeated-measurements ANOVA on maximal angles (Table 2) and the functional F test for the swing phase (Table 3). Overall, the results from the different approaches seem to inform each other.

## Discussion

We collected time-dense gait data for 28 lower limbs in 14 children with cerebral palsy, typically hemiplegia, in a repeated-measurements design where every individual was measured while walking both barefoot and shod with ankle-foot orthoses. Gait curves depicting rotations for lower limb segments in different planes were modeled by a functional mixed-effects model. The data were analyzed by using three different methods: multiple testing with pointwise F tests performed at separate points of a fine grid, a new functional F test which uses entire gait curves, and univariate repeated-measurements ANOVA, which was performed separately on minimum and maximum rotations. The results obtained by these approaches had many fundamental commonalities, but there were also some differences which warrant further explanation. In what follows, we first interpret the results and discuss some limitations of the approach and the current study. We then discuss some methodological issues and extensions of the proposed functional F test to more complicated experimental designs.

Biomechanical effects of ankle-foot orthoses, including direct effects to the limb segments contained within the orthoses and indirect effects to the rest of the body, mainly shank kinematics^27^, are consistent with the results from functional F tests. Moreover, ankle-foot orthoses can be seen to have a greater effect on sagittal joint rotations as compared to coronal and horizontal planes. This is likely due to the design of bespoke ankle-foot orthoses for patients with cerebral palsy (Fig. 1). The rigid L shaped ankle-foot orthoses with an upright portion behind the calf greatly limits plantar flexion and dorsiflexion of the ankle and foot. Moreover, the distal anterior ankle strap and the foot plate have a joint fixing effect which is associated with decreased orthogonal plane rotations. Thus, effects on ankle rotation in the coronal plane and foot rotation in the horizontal plane, which were detected by the functional F tests, are possibly due to some compensatory mechanism.

There are some limitations to this study. One of the issues we debated at length was that of using each leg of each subject as an independent observation. The literature is divided on this issue^42^, however we felt this was justified because the between limb correlation was low, subjects typically had hemiplegia and therefore moved asymmetrically and the purpose of the study was to compare different models rather than make definitive clinical or applied scientific recommendations. Moreover, depending on severity of spasticity, children with cerebral palsy are prone to fatigue after short bouts of low to medium intensity activity^43^. In order to minimize patient fatigue and to maximize data output, patients were asked to walk barefoot and without walking aids, if possible, before walking with ankle-foot orthoses. As a consequence, the effect of wearing/not wearing ankle-foot orthoses is confounded with a potential effect of the walking condition testing order. To strengthen the study design, the order of walking conditions should be randomized to eliminate or reduce potential systematic biases^44–47^, if possible.

Another limitation of this study is that we only studied a sample of 14 patients. While this is considered small for many statistical applications, it is not uncommon for studies of this type, in this patient population, to have similar sample sizes^48^. This issue has been commonly addressed in gait study reports investigating cerebral palsy^49–51^ and is mainly due to the restricted inclusion criteria necessary for enrolment, necessary due to the extremely heterogeneous nature of movement impairments in people with cerebral palsy^51^. Other studies to assess the effects of ankle-foot orthoses in patients with cerebral palsy^22–24, 52–61^ have used sample sizes that are similar to that of the current study, and our approach and findings are therefore justifiable and comparable respectively. Furthermore, in the present study we examined retrospective data but have not explored clinical patient information (i.e. gait type, severity of spasticity). Our focus was to quantitatively validate the functional mixed-effects ANOVA as a means of determining differences in gait between barefoot walking versus the use of ankle-foot orthoses in a relatively homogeneous but clinically relevant patient group. Therefore, at this stage we have excluded detailed clinical discussion.

We now give possible reasons why there are sometimes discrepancies between the statistical results. Differences between the functional F tests and the repeated-measurements ANOVA for minimal and, respectively, maximal angles may arise from the fact that the former tests consider the whole gait cycle, whereas the repeated-measurements ANOVA provides a univariate analysis in which the values of the response variable correspond to the most extreme observations that occur throughout the gait cycle. These extremes, e.g. minimal angles, occur at points of the gait cycle that vary within and between patients. For example, for the hip joint in the sagittal plane we examined the data and found that for most patients and most walks minimal angles occurred well before the end of the stance phase, but there were also two patients for whom minimal angles occurred during the swing phase.

Regarding differences in the results between the pointwise F tests and the repeated-measurements ANOVA, we note that with respect to comparing the conditions of wearing and not wearing ankle-foot orthoses every single pointwise F test as well as the repeated-measurements F test is mathematically equivalent to a standard paired t test^62^ on 28 pairs of observations, where every pair consists of observations for a single leg that is observed with and without orthoses. If, for example, the minimal angle always occurred at the same time point, then the F test of the repeated-measurements ANOVA on minimal angles would coincide with the pointwise F test at this particular point in time. However, since minimal angles occur at different time points, results from both tests will be different. Put differently, although all pointwise F tests and the repeated-measurements F test use the same formula for calculating the test statistic, the tests apply this formula to different data with a difference in results of findings.

Differences in the results of pointwise F tests and functional F tests may be caused by the fact that the familywise error probability^63^ for the whole or parts of the gait cycle of the multiple testing approach exceeds the nominal significance level of the individual pointwise tests. One way to alleviate this problem would be to apply a Bonferroni correction to the nominal significance level of the pointwise F tests. If this were to be done for the whole gait cycle, each of those tests would need to use a significance level that was equal to, for instance, 0.05 divided by the number of tests. In the current study, we used 201 separate pointwise tests and hence, in Fig. 4, the critical value represented by the line would need to be adjusted from ${F}_{0.05}\mathrm{(1},\mathrm{27)}=4.21$ to ${F}_{\mathrm{0.05/201}}\mathrm{(1},27)=17.78$ ^64^ with the consequence that only the effects in the sagittal plane on the ankle joint and foot would remain significant. Thus the Bonferroni correction would be overly conservative which is one reason why it is not recommended in the FDA literature^65^. Notwithstanding, some adjustment of the nominal significance level that is used for the pointwise F tests would seem to be appropriate in order to avoid too many type I errors. Although not designed for this purpose, the functional F tests appear to achieve this goal by integrating information over the whole or parts of the gait cycle while maintaining the pre-specified significance level.

We believe that the different types of analysis considered in this paper should be regarded as being complementary rather than competing. Although we would not recommend pointwise testing alone, we nevertheless find this approach useful since from results like those in Fig. 4 one can see very easily where in the gait cycle effects occur. However, we think of pointwise tests as more of an exploratory rather than a confirmatory tool, so when reporting an effect as being significant we would prefer to base this decision on a functional F test. With respect to detecting effects in specific parts of the gait cycle very little seems to be lost by this approach, since, as shown in Table 3, the functional F test can be flexibly applied to different phases of the gait cycle. Analyzing specific gait features, like the minimum and maximum angles in the current study, may also be useful but we believe this type of analysis should be motivated by biomechanical considerations or specific clinical questions and not be used for the reason that it sidesteps the difficult analysis of entire curves.

The functional mixed-effects ANOVA model in this paper and the method for obtaining functional F tests can be generalized to more complex experimental designs in which, like in the present study, there is correlation between entire curves. More precisely, the methodology can be applied to experiments with an orthogonal block structure^66–68^ and to general orthogonal designs^47^. These designs include, for example, randomized complete block designs, row-column and split-plot designs. Current treatments^3^ of testing problems for functional ANOVA models appear to only consider experiments whose layout is given by a completely randomized design.

Further validation, including structured clinician and patient engagement, is warranted to clarify whether our interpretation of the individually or collectively applied statistical analyses in this paper adds value in practice. Ultimately, the litmus test of whether this novel statistical analysis is truly useful would be improved patient outcomes, a subject for future work.

### Data Availability

The datasets generated and analysed during the current study are available from the corresponding author on reasonable request.
